# Novel indolin-2-one-substituted methanofullerenes bearing long *n*-alkyl chains: synthesis and application in bulk-heterojunction solar cells

**DOI:** 10.3762/bjoc.10.111

**Published:** 2014-05-14

**Authors:** Irina P Romanova, Andrei V Bogdanov, Inessa A Izdelieva, Vasily A Trukhanov, Gulnara R Shaikhutdinova, Dmitry G Yakhvarov, Shamil K Latypov, Vladimir F Mironov, Vladimir A Dyakov, Ilya V Golovnin, Dmitry Yu Paraschuk, Oleg G Sinyashin

**Affiliations:** 1 A.E. Arbuzov Institute of Organic and Physical Chemistry Kazan Research Center of the Russian Academy of Sciences, Kazan 420088, Russian Federation; 2 Faculty of Physics and International Laser Center, M.V. Lomonosov Moscow State University, Moscow 119991, Russian Federation; 3 Faculty of Bioengineering and Bioinformatics, M.V. Lomonosov Moscow State University, Moscow 119991, Russian Federation

**Keywords:** bulk heterojunction, fullerenes, isatin, phosphorus, photovoltaics

## Abstract

An easy, high-yield and atom-economic procedure of a C_60_ fullerene modification using a reaction of fullerene C_60_ with *N*-alkylisatins in the presence of tris(diethylamino)phosphine to form novel long-chain alkylindolinone-substituted methanofullerenes (AIMs) is described. Optical absorption, electrochemical properties and solubility of AIMs were studied. Poly(3-hexylthiophene-2,5-diyl) (P3HT)/AIMs solar cells were fabricated and the effect of the AIM alkyl chain length and the P3HT:AIM ratio on the solar cell performance was studied. The power conversion efficiencies of about 2% were measured in the P3HT/AIM devices with 1:0.4 P3HT:AIM weight ratio for the AIMs with hexadecyl and dodecyl substituents. From the optical and AFM data, we suggested that the AIMs, in contrast to [6,6]-phenyl-C_61_-butyric acid methyl ester (PCBM), do not disturb the P3HT crystalline domains. Moreover, the more soluble AIMs do not show a better miscibility with the P3HT crystalline phase.

## Introduction

Organic photovoltaics are a rapidly growing field of research due to its potential for production of low-cost and flexible solar cells [1–3]. Organic solar cells are mainly based on bulk heterojunctions composed of a polymer donor and a fullerene acceptor. A number of promising donor materials has been developed, whereas very few successful fullerene derivatives have been proposed. The most popular fullerene derivative in organic photovotaics is (PCBM) [4–6]. As an alternative to PCBM, we recently suggested indolinone-substituted methanofullerenes (IM) [7]. The principal advantages of IM are their easier synthesis and purification as compared to PCBM [7–8]. Indeed, the IM can be synthesized by reaction of fullerene with isatins in the presence of tris(diethylamino)phosphine, and the reaction requires neither heat nor irradiation and results in only the 6,6-closed monoadduct with high yield. In addition, the IM chemical structure can be varied by substitutions at the nitrogen atom and/or aromatic ring to achieve the required solubility and miscibility with the donor materials in the bulk heterojunction.

**Figure 1:**
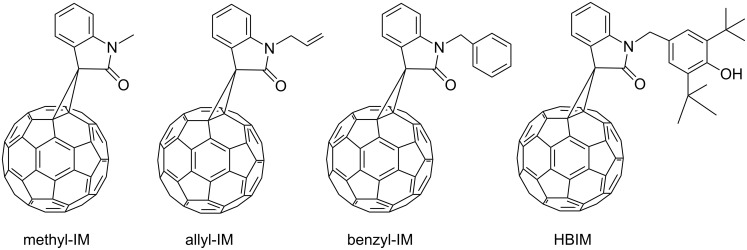
Structures of the indolinone-substituted methanofullerenes prepared earlier.

Earlier we described the synthesis of IM having methyl- (**AIM 1**), allyl- (allyl-IM) or aryl- (benzyl-IM) substituents at the nitrogen atom (Figure 1) [7,9]. The first IM-containing plastic solar cells (PSCs) were fabricated on the P3HT/HBIM blends, the power conversion efficiency (PCE) was about 2% [8]. The P3HT/HBIM devices showed the same open-circuit voltage (*V*_oc_) as the P3HT:PCBM ones, but the short-circuit current (*J*_sc_) and the fill factor (*FF*) were considerably less. It was suggested that the coarser surface morphology in the P3HT**/**HBIM blends is a result of the fullerene-rich domains, which are too large for the optimal polymer–fullerene phase separation. Possibly, the coarser surface morphology is due to the lower solubility of HBIM as compared to PCBM.

Usually, alkyl fragments are used to increase the fullerene solubility in organic solvents so that the longer the fragment the higher the solubility. For example, this approach was used to increase the solubility of indole-substituted fullerenopyrrolidines [10]. In this work, we increase the IM solubility by introduction of long-chain alkyl fragments to the IM nitrogen atom. As a result, the series of novel alkyl-containing indolin-2-one-substituted methanofullerenes (**AIMs 2**–**9**) were synthesized. Optical absorption and electrochemical properties of **AIMs 1**–**9** were studied. Bulk heterojunction polymer solar cells based on P3HT/AIM blends were fabricated and characterized.

## Results and Discussion

### Synthesis of **AIMs 1**–**9**

**Scheme 1:**
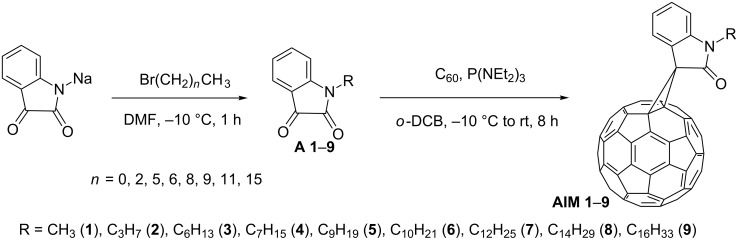
The two-step synthetic pathway towards the methanofullerenes **AIM 1**–**9**.

The reaction for the synthesis of IM allows us to obtain a wide range of various IM [7,9]. The variation of IM is easy carried out by the variation of pristine indoline-2,3-diones (isatins). Thus the series of *N*-alkylisatins **A 1**–**9** were used for the synthesis of **AIMs 1**–**9** (Scheme 1). The isatins **A 1**–**9** were obtained by reaction of isatin sodium salt with the corresponding *N*-bromoalkanes [11–14].

The reactions of **A 1**–**9** with fullerene C_60_ and tris(diethylamino)phosphine were conducted in *o*-dichlorobenzene (*o*-DCB) at −10 °C followed by warming-up to room temperature. The separation of the reaction products by column chromatography on silica gel gave the unreacted fullerene, corresponding **AIMs 1**–**9**, and polyadduct mixtures. The yields of **AIMs 1**–**9** were within 30–48% with respect to the starting fullerene. UV–vis, IR, and NMR spectroscopy confirmed their structure. The composition was established by MALDI–TOF mass spectrometry.

### Optical absorption, electrochemical properties and solubility of AIMs

**Figure 2:**
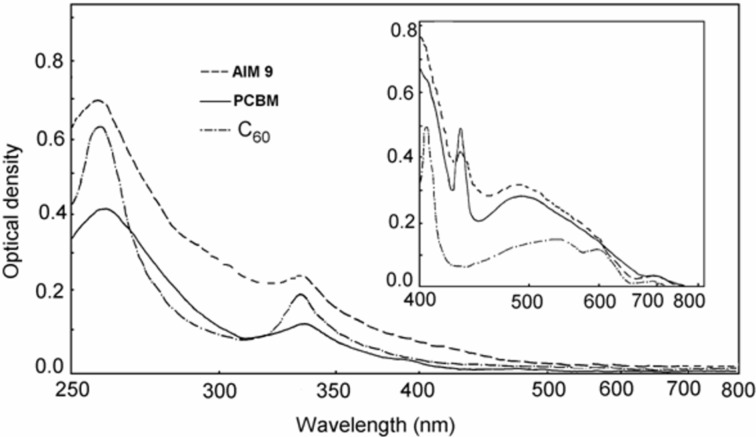
Optical absorption spectra of **AIM 9,** PCBM, and C_60_ in CH_2_Cl_2_ (2·10^−5^ mol·L^−1^, the cell thickness *d* = 2 mm). Inset shows the absorption spectra at *d* = 10 mm.

Figure 2 compares a typical optical absorption spectrum of AIMs with those of PCBM and pristine fullerene C_60_. In the UV region from 200 to 350 nm, AIMs are characterized by two absorption peaks as PCBM and fullerene, which are typical for π–π* transitions in aromatic systems. In the visible region, a characteristic band near 428 nm for 6,6-fullerene mono-cycloadducts is observed (Figure 2, inset). Note that, for the wavelengths shorter than 600 nm, the AIM absorption is stronger than that of PCBM and fullerene C_60_ that could be assigned to the effect of the indolinone fragment.

**Figure 3:**
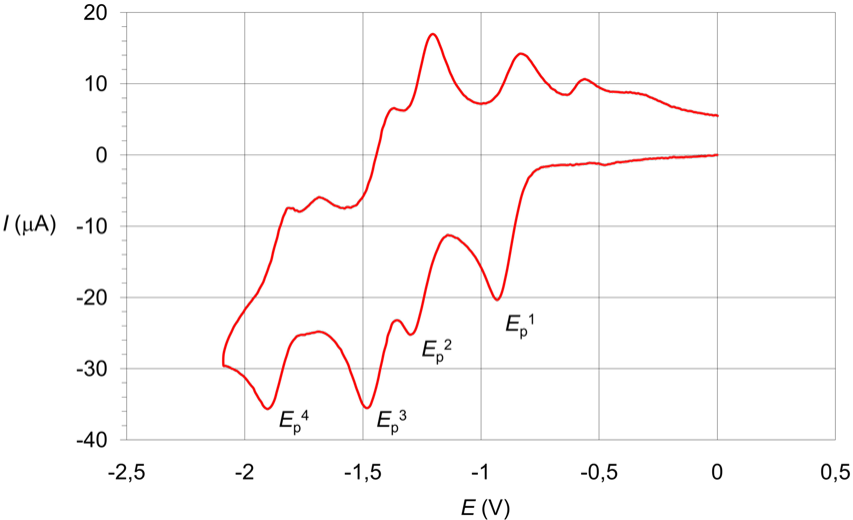
Cyclic voltammetry curve of **AIM 9**.

To evaluate the stability of reduced AIMs and the AIM LUMO energy levels, a CV study was performed. Four reversible peaks are observed in the CV data of **AMF 1**–**9**. Table 1 summarizes the results, and Figure 3 presents a typical CV curve of AIM. Note that both the fullerene sphere and the indolinone fragment are electrochemically active in the working electrochemical window. Thus, the pristine fullerene C_60_ and isatins **A 1**–**9** are reduced in the potential range from 0 to –2.5 V. A comparison of the peak potentials of the first reduction process of C_60_, **A 1**–**9** and **AIM 1**–**9** allows us to conclude that the electron transfer onto the fullerene sphere is the initial stage of the AIM electroreduction. The other cathodic peaks correspond to the reduction of both the indolinone fragments and the fullerene sphere [7,15]. First peaks of all the **AIM 1**–**9** are shifted to the more cathodic potentials in comparison with that for C_60_. The electrochemical data indicate that AIM could be attractive acceptors for fabrication of PSCs.

**Table 1:** Peak potentials^a^ of C_60_, **AIM 1**–**9** and **A 1**–**9** and LUMO energy levels of **AIM 1**–**9** and PCBM.

Compd	*E*p^1^ (V)	*E*p^2^ (V)	*E*p^3^ (V)	*E*p^4^ (V)	LUMO (eV)

C_60_	−0.83	−1.24	−1.70	−2.16	−3.77

PC_60_BM					−3.67 ref. [8]
**AIM 1**	−0.89	−1.22	−1.43	−1.85	−3.71
**AIM 2**	−0.86	−1.23	−1.42	−1.87	−3.74
**AIM 3**	−0.93	−1.29	−1.47	−1.90	−3.67
**AIM 4**	−0.86	−1.24	−1.42	−1.84	−3.74
**AIM 5**	−0.89	−1.22	−1.41	−1.84	−3.71
**AIM 6**	−0.91	−1.25	−1.44	−1.86	−3.69
**AIM 7**	−0.89	−1.26	−1.46	−1.88	−3.71
**AIM 8**	−0.90	−1.24	−1.44	−1.88	−3.70
**AIM 9**	−0.94	−1.30	−1.48	−1.91	−3.66
**A 1**	−1.35	−2.02			
**A 2**	−1.37	−2.04			
**A 3**	−1.39	−1.99			
**A 4**	−1.31	−1.99			
**A 5**	−1.39	−2.06			
**A 6**	−1.34	−1.99			
**A 7**	−1.33	−1.99			
**A 8**	−1.33	−2.04			
**A 9**	−1.38	−2.03			

^a^Potential values are mentioned vs Ag/Ag^+^ reference electrode.

Chlorobenzene is a typical solvent for preparation of PSCs. It is believed that the optimal solubility of fullerene derivatives in chlorobenzene is in the range 30–80 mg·mL^−1^ at room temperature [16]. It seems that this range of fullerene solubility is needed to provide the optimal bulk heterojunction morphology, in which the typical size of the phase-separated domains is about tens of nanometers. The optimal morphology supports efficient charge separation and charge transport in bulk heterojunction solar cells. Our studies showed that the increasing of the alkyl chain length from C1 to C9 had not an important influence on the AIM solubility, the solubility of **AIMs 1**–**5** was 3–5 mg·mL^−1^. **AIMs 6**–**8** have a higher solubility, and it was 10 mg·mL^−1^ for **AIM 6** and 20 mg·mL^−1^ for **AIMs 7**,**8**. The solubility of **AIM 9** was even higher 40 mg·mL^−1^. Therefore, one may expect that the long-chain AIMs are promising acceptors for PSCs as they have the higher solubility. To evaluate the real potential of AIMs**,** PSCs were fabricated and studied.

### Solar cells

**Figure 4:**
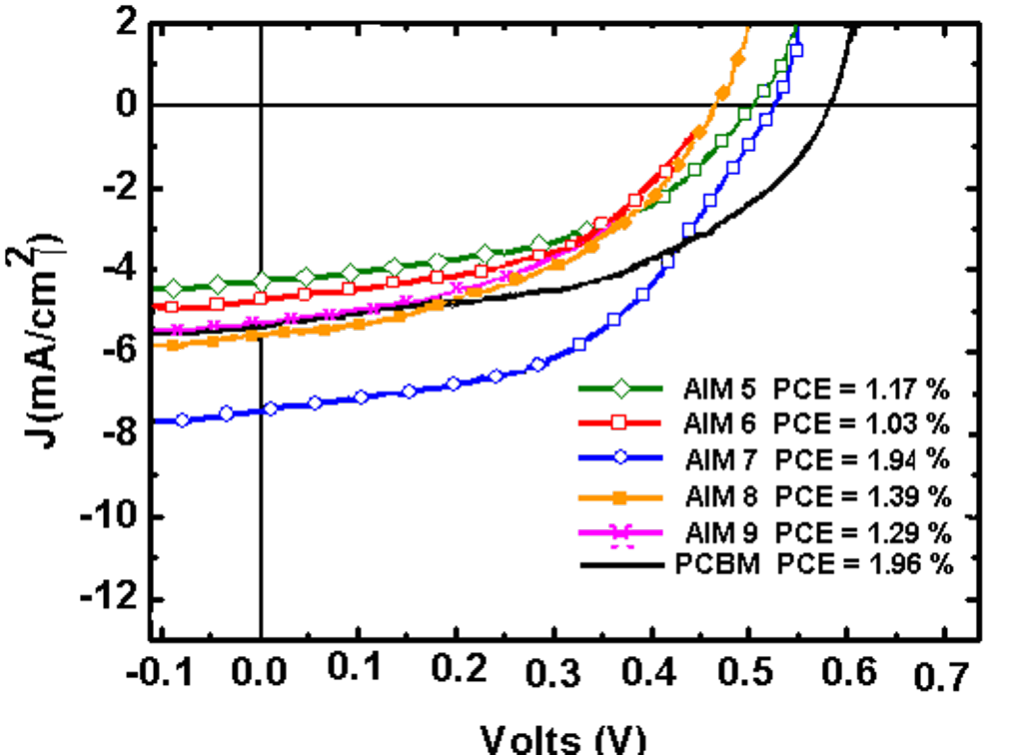
*J*–*V* characteristics of P3HT/AIMs and reference P3HT/PCBM devices for the P3HT/fullerene weight ratio 1:1.

We fabricated P3HT-fullerene solar cells by using only **AIM 5**–**9** as the solubility of **AIM 1**–**4** in chlorobenzene is less than 5 mg/mL, and this solubility is too low for efficient polymer bulk heterojunction solar cells [16]. DCB was used as a solvent as the AIM solubility in DCB is higher than in chlorobenzene. The solar cells were fabricated according to the standard protocol for P3HT/PCBM devices excluding thermal annealing. Figure 4 presents current-density–voltage (*J*–*V*) characteristics of the P3HT/**AIM 5**–**9** devices for the polymer/fullerene weight ratio 1:1. This ratio is usually considered to be close to the optimal one for various polymer/fullerene solar cells. The data for the P3HT/PCBM reference solar cell are also shown for comparison. The performance of polymer/fullerene solar cells is known to depend on the polymer/fullerene ratio and on the post-treatment conditions, specifically on thermal annealing. For **AIM 7**,**9**, we performed a more detailed study varying the polymer/fullerene ratio from 1:0.2 to 1:1.8 and the thermal annealing conditions. Table 2 summarizes the photovoltaic parameters of the P3HT/**AIM 5**–**9** devices, i.e., the short-current density (*J_SC_*), open-circuit voltage (*V_OC_*), fill factor (*FF*), and power conversion efficiency (PCE).

**Table 2:** Photovoltaic parameters of the polymer/fullerene solar cells.

Active layer	Weight ratio	Thermal annealing	*J*_SC_/mA cm^−2^	*V*_OC_/V	*FF*	*PCE*/%

P3HT/**AIM 5**	1 : 1	–	4.30	0.55	0.55	1.17
P3HT/**AIM 6**	1 : 1	–	4.89	0.48	0.45	1.03
P3HT/**AIM 7**	1 : 0.2	–	3.78	0.50	0.45	0.85
	1 : 0.4	–	6.80	0.54	0.55	**2.03**
	1 : 0.6	–	7.44	0.51	0.52	1.96
	1 : 0.8	–	7.94	0.53	0.44	1.83
	1 : 1	–	7.60	0.55	0.47	1.94
	1 : 1.5	–	7.65	0.55	0.51	1.99
	1 : 1.5	5 min, 30 °C	6.48	0.54	0.44	1.39
	1 : 1.5	5 min, 60 °C	7.28	0.45	0.44	1.37
	1 : 1.5	5 min, 90 °C	7.59	0.43	0.34	1.05
	1 : 1.5	5 min, 120 °C	2.52	0.32	0.25	0.20
	1 : 1.8	–	6.28	0.64	0.55	1.97

P3HT/**AIM 8**	1 : 1	–	5.94	0.48	0.54	1.39
	1 : 1	5 min, 50 °C	5.87	0.37	0.53	1.14

P3HT/**AIM 9**	1 : 0.2	–	3.29	0.51	0.46	0.77
	1 : 0.4	–	7.23	0.54	0.57	**2.09**
	1 : 0.6	–	6.17	0.45	0.57	1.54
	1 : 0.8	–	5.84	0.51	0.47	1.33
	1 : 1	–	5.45	0.50	0.54	1.29
	1 : 1	5 min, 60 °C	3.81	0.66	0.49	0.95
	1 : 1.5	–	4.42	0.54	0.53	1.07
	1 : 1.8	–	3.94	0.45	0.53	0.81

P3HT/PCBM	1 : 1	–	5.44	0.59	0.62	1.96

First of all, the data for the non-annealed devices should be considered. For the AIM-containing devices, the typical *V_oc_* was in the range 0.5–0.6 V and was close to that of the reference P3HT/PCBM solar cell (*V*_oc_ = 0.59 V). The *FF* was higher than 50% for the best devices with the maximum value 57% (Table 2) that was somewhat lower than that of the reference P3HT/PCBM solar cell (*FF* = 62%).

For the low content of **AIM 7**,**9** in the blend (1:0.2), the solar cells showed the PCEs below 1% mainly because of low *J*_sc_ (3.8 and 3.3 mA/cm, correspondingly). The low content of fullerene acceptor is insufficient for efficient charge transport in the bulk heterojunction as was observed in many other polymer–fullerene solar cells. The best performance for the P3HT/AIM devices was observed for **AIM 7**,**9** with polymer/fullerene ratio 1:0.4 for which the PCE was 2.03 and 2.09%, correspondingly. This rise in the PCE is mainly due to increase in *J*_sc_ from 3.8 to 6.8 mA/cm^2^ for **AIM 7** and from 3.3 to 7.2 mA/cm^2^ for **AIM 9**. In addition, the *FF* increased from 45% to 55% and from 46% to 57% for **AIM 7**,**9**, respectively. Further increase of **AIM 7** content in the blend does not considerably change the photovoltaic parameters so that the PCE was around 2%. In contrast, increasing the **AIM 9** content higher than 1:0.4 led to a gradual decrease in the PCE mainly due to a decrease in the *J*_sc_.

**Figure 5:**
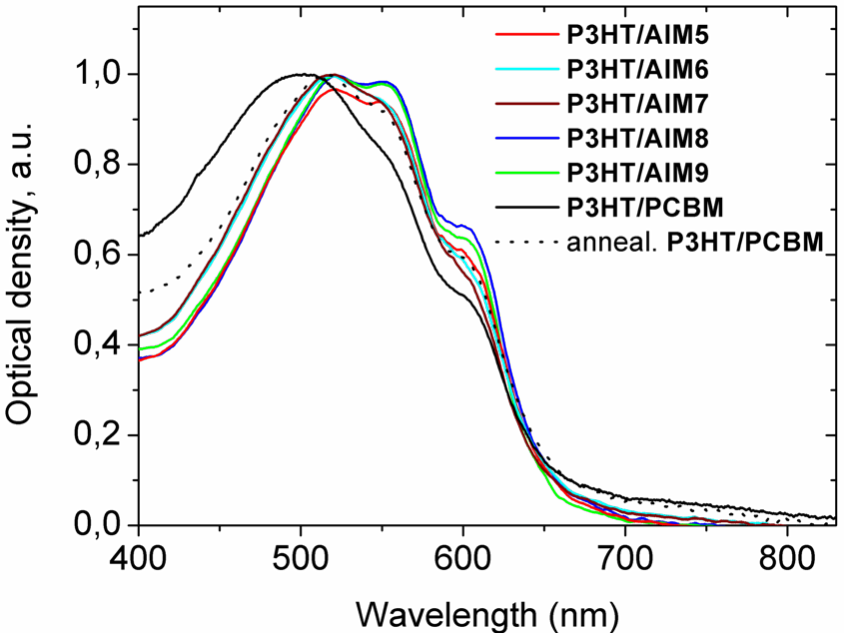
Absorption spectra of P3HT/fullerene blended films. The P3HT/PCBM blend was annealed during 15 min at 130 °C.

**Figure 6:**
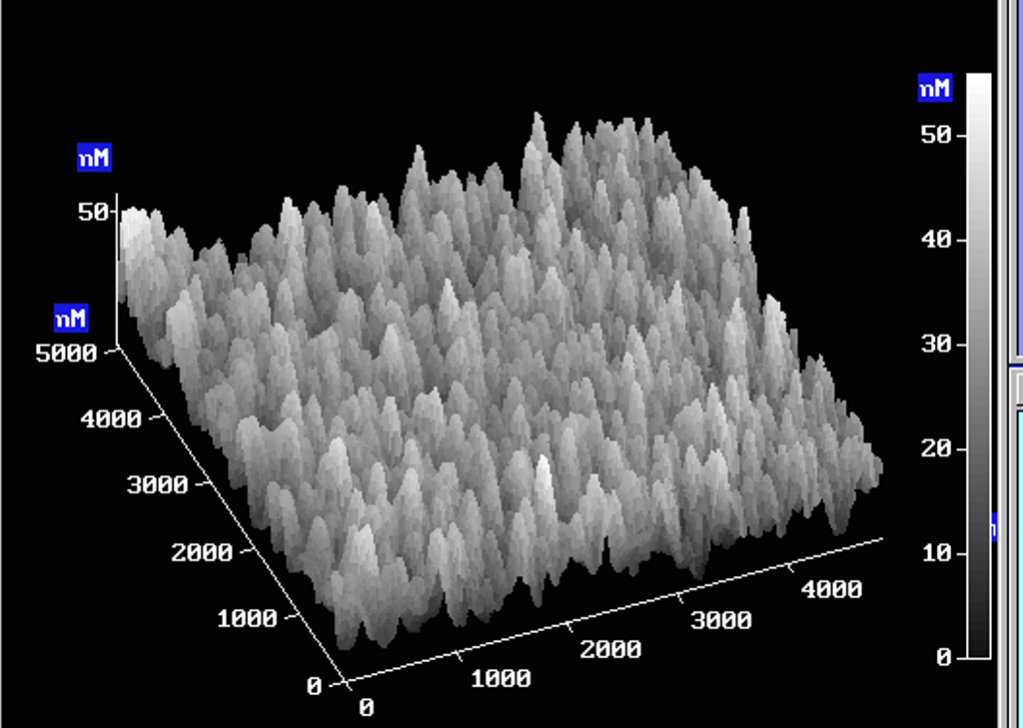
AFM topography image of an as-casted 1:1 P3HT:**AIM 7** blended film.

In contrast to P3HT/PCBM solar cells all the photovoltaic parameters of P3HT/AIMs generally decreased after thermal annealing (see Table 2). Even 5 min annealing at temperatures below 60 °C degraded the device performance. To reveal the effect of thermal annealing in P3HT/AIM blends, we recorded optical absorption spectra of P3HT/**AIM 5**–**9** and P3HT/PCBM blended films (Figure 5). The fine structure of the P3HT absorption edge (550–650 nm) is due to the P3HT crystalline phase [17]. As follows from Figure 5, the as-cast P3HT/PCBM film has the weakest features at 550 and 600 nm indicating the lowest content of the P3HT crystalline phase. This was assigned to disorder in the P3HT crystalline phase induced by the PCBM molecules [18]. Thermal annealing of the P3HT/PCBM blend increases the content of the P3HT crystalline phase (the black curve in Figure 5, for which the features at 550 and 600 nm are more pronounced) so that more or less optimal morphology in the P3HT/PCBM blend is achieved. However, according to Figure 6, the content of the P3HT crystalline phase in the as-prepared P3HT/AIM blends is already no less than in that in the optimized P3HT/PCBM blend. Indeed, the absorption at 550 and 600 nm is higher for P3HT/**AIM 8**,**9** than in the annealed P3HT/PCBM blend showing that the content of the polymer crystalline phase is higher than optimal. From these data, one can suggest that the AIM miscibility with the P3HT crystalline phase is far less than that of PCBM. Possibly, the blends of P3HT with the long-chain alkyl AIMs contain relatively large polymer crystalline domains without the fullerene phase, which do not work efficiently in the photoinduced charge separation and collection. This could explain the modest performance of P3HT/AIMs solar cells as compared with P3HT/PCBM ones and the negative effect of thermal annealing. As a result, according to the optical data, the higher AIM solubility does not provide the better miscibility between the AIM and the P3HT crystalline phase. This is in agreement with the AFM data (see below). Therefore, one can conclude that increasing the fullerene solubility does not always improve the polymer–fullerene miscibility.

Another interesting feature in Figure 5 is the difference in optical absorption of the AIM and PCBM in the range 600–800 nm. The optical absorption of P3HT/fullerene blends in this spectral range is known due to fullerene aggregates [19]. As Figure 5 shows, the optical absorption of the AIMs is considerably lower than that of PCBM. Therefore, one can suggest the lower tendency to aggregation of the AIMs. This may be explained by the bulkier indolinone addend as compared to those in common methanofullerenes. The lower aggregation tendency could be an additional factor that influences the morphology in polymer/AIM blends. Moreover, the lower AIM aggregation could result in less efficient electron transport in the AIM domains. Lower electron mobility in the AIM domains could result in unbalanced transport of electrons and holes in the active layer and space charge formation, which decreases the photocurrent and PCE [20].

The surface of the P3HT/AIM blended films was studied by atomic-force microscopy (AFM). Generally, the P3HT/**AIM 7**–**9** films showed a phase separated morphology with a characteristic domain height in the range of 10–40 nm similar to the standard P3HT/PCBM films. Figure 6 shows a typical AFM image of an as-casted P3HT**/**AIM film. However, in contrast to P3HT/PCBM films, we did not observe any clear correlations between the morphology features and the AIM content in films with the AIM content higher than 0.4. Moreover, we did not find any distinct relation between the AIM solubility and the morphology in P3HT/**AIM 7**–**9** blends. From these observations, one could suppose that the relation between the AIM solubility and its miscibility with P3HT is not straightforward. This is in accordance with optical absorption data discussed above.

As mentioned above, the AIM solubility increases in the series from **AIM 5** to **AIM 9**. As follows from Table 2, increasing the AIM solubility from **AIM 5** to **AIM 7** results in enhanced device performance, whereas for **AIM 7**–**9** no clear correlation between the solubility and the device performance is observed. As discussed above, the AIM solubility increase from **AIM 7** to **AIM 9** does not improve the miscibility of the AIM with the P3HT crystalline phase. Moreover, lengthening the AIM alkyl chain could impede the electron transport in the AIM phase of the bulk heterojunction. This could explain that the PCE of the best devices with **AIM 7**,**9** is almost the same despite the solubility of **AIM 9** is higher than **AIM 7**.

## Conclusion

A series of new long-chain alkyl indolinono-substituted methanofullerenes were synthesized by reaction of fullerene C_60_ with *N*-alkylisatins having nine different *n*-alkyl substituents. Their optical absorption and the first electrochemical reduction potential are close to those of PCBM. The lengthening of the *n*-alkyl radical at the nitrogen atom results in a gradual increase in the AIM solubility. The photovoltaic properties of PSCs based on the five AIMs with the longest alkyl chains were studied. The standard fabrication protocol optimized for P3HT/PCBM solar cells was used excluding thermal annealing post-treatment. The power conversion efficiencies of about 2% were measured in the P3HT/AIM devices with hexadecyl and dodecyl substituents. Thermal annealing was observed to be not efficient post-treatment for P3HT/AIM solar cells. From the optical and AFM data, we suggested that the AIMs, in contrast to PCBM, do not disturb the P3HT crystalline domains. Moreover, the more soluble AIMs do not show better miscibility with the P3HT crystalline phase so that no clear correlation between the AIM solubility and its miscibility with P3HT was observed. We suppose that fine tuning of the fabrication protocol is needed to reveal the potential of AIM for polymer/fullerene solar cells.

## Experimental

### Synthesis

1-Methyl-3-(3-cyclopropane[1,9](C60-*I*_h_)[5,6]fulleren-3-yl)-indolin-2-one (**AIM 1**) was synthesized according to ref. [7]. Isatins **A 2**–**9** were obtained by known procedures [11–14].

The general procedure for synthesis of 1-alkyl-3-(3-cyclopropane[1,9](C_60_-*I*_h_)[5,6]fulleren-3-yl)-indolin-2-one (**AIMs 2**–**9**) was as follows: tris(diethylamino)phosphine (0.1 g, 0.4 mmol) was added dropwise to a mixture of the corresponding isatins **A 2**–**9** (0.14 mmol) and C_60_ (0.1 g, 0.14 mmol) in anhydrous *o*-dichlorobenzene (40 mL) at –10 °С. The mixture was stirred for 8 h at –10 °С and then the temperature was allowed to rise to room temperature. The solvent was removed under reduced pressure and the residue was purified by column chromatography on silica gel using a mixture of toluene and petroleum ether 4:5 as eluent. After elution of the recovered C_60_, the fraction containing the desired compound was collected and dried in vacuo at 30–40 °C for 6 h.

Analytical data of these compounds can be found in Supporting Information File 1.

### Materials, characterization and devices

P3HT (Rieke Metals), PCBM (Solenne), poly(3,4-ethylenedioxythiophene):poly(styrene sulfonate), PEDOT:PSS (Baytron P VP AI 4083, H.C. Stark) and 1,2-dichlorobenzene (DCB) were used as received.

IR spectra were recorded using a Bruker IFS-113V instrument. UV–vis spectra in solution were recorded using a Specord UV–vis spectrophotometer. Absorption spectra in films were recorded with the help of a fiber-coupled spectrophotometer (Avantes). Mass-spectra were recorded with the use of a «Bruker Ultraflex III MALDI TOF/TOF SYSTEM» apparatus. NMR experiments were carried out with a Bruker AVANCE-600 spectrometer (14.1 T) equipped with a pulsed gradient unit capable of producing magnetic field pulse gradients in the z-direction of 56 G·cm^−1^. All spectra were acquired in a 5 mm inverse probehead working at 600.0 MHz in ^1^H and 150.9 MHz in ^13^C experiments. Chemical shifts are reported on the δ (ppm) scale and are relative to the residual ^1^H and ^13^C signal of CDCl_3_.

The cyclic voltammetry (CV) curves were recorded using a three-electrode type electrochemical cell in *o*-DCB/MeCN (3:1 by volume) solution in the presence of Bu_4_NBF_4_ (0.1 M) with a potential sweep rate of 50 mV·s^–1^ with the help of a PI-50-1 potentiostat. A silver electrode Ag/AgNO_3_ (0.01 *M* solution in MeCN) was served as the reference electrode (*E°*(Fc/Fc^+^) = +0.20 V). A glassy carbon (GC) electrode with a working surface of 3.14 mm^2^ was served as the working electrode and a Pt wire with a diameter of 1 mm was used as the auxiliary electrode. Measurements were carried out at thermostatic conditions (20 °C) in nitrogen atmosphere. The concentration of the substrate was 5 10^−3^ M. The LUMO energy levels of the fullerene derivatives were calculated from the equation: LUMO = −(*E_p_*^red(1)^ – *E^1^_ferrocene_* + 4.8), where *E_p_*^red(1)^ is the potential of the first reduction peak with unit of V vs. Fc/Fc^+^ [21].

Solar cells with the device configuration ITO/PEDOT:PSS/active layer/CaAl were fabricated on ITO-coated glasses using a spin-coated procedure (at 3000 rpm for 2 min for PEDOT:PSS layer and at 1000 rpm for 2 min for active layer which was either the mixture of P3HT and AIM or P3HT and PCBM in DCB). The total concentration of the active layer components was 20 g/L. Alloy of calcium and aluminum CaAl (~100 nm) was used as the low-work-function electrode and was thermally deposited on the active layer through a shadow mask with a pixel area of 3.8 mm^2^ (8 pixels per one sample). The devices were thermal treated on a hotplate in an argon-filled glove box. The solar cells efficiencies were calculated from current–voltage (*J–V*) curves which were obtained using a computer-controlled Keithley 2400 SourceMeter instrument. The devices were illuminated through a 3.2 mm^2^ circular aperture, which was in contact with the glass side of the device, by using a 150 W solar simulator (model 9600, Newport) with an AM1.5G filter, the optical power on the sample was set to about 100 mW/cm^2^ with the help of a bolometric detector. Atomic force microscopy (AFM) images of the polymer-fulllerene blended films were recorded with a Smena instrument (NT-MDT) in the tapping mode.

## Supporting Information

File 1 Analytical data of **AIM 2–9**.
