# Supplementary material for: Novel indolin-2-one-substituted methanofullerenes bearing long n-alkyl chains: synthesis and application in bulk-heterojunction solar cells
Source: Beilstein J Org Chem. 2014 May 14;10:1121–8. doi: 10.3762/bjoc.10.111 (PMC4077430; doi:10.3762/bjoc.10.111)
Supplement: File 1 — Analytical data of AIM 2–9. [file Beilstein_J_Org_Chem-10-1121-s001.pdf]

Supporting Information

for

Novel indolin-2-one-substituted methanofullerenes bearing long *n*-alkyl chains: synthesis and application in bulk-heterojunction solar cells

Irina P. Romanova[1], Andrei V. Bogdanov*[1], Inessa A. Izdelieva[2,3], Vasily A. Trukhanov[2], Gulnara R. Shaikhutdinova[1], Dmitry G. Yakhvarov[1], Shamil K. Latypov[1], Vladimir F. Mironov[1], Vladimir A. Dyakov[2,] Ilya V. Golovnin[2], Dmitry Yu. Paraschuk[2] and Oleg G. Sinyashin[1]

Address: [1]A.E. Arbuzov Institute of Organic and Physical Chemistry Kazan Research Center of the Russian Academy of Sciences, Kazan 420088, Russian Federation, [2]Faculty of Physics and International Laser Center, M.V. Lomonosov Moscow State University, Moscow 119991, Russian Federation and [3]Faculty of Bioengineering and Bioinformatics, M.V. Lomonosov Moscow State University, Moscow 119991, Russian Federation

Email: Andrei Bogdanov - abogdanov@inbox.ru

*Corresponding author

Analytical data of AIM 2–9

1-*n*-Propyl-3-(3-cyclopropane[1,9](C_{60}-I_h)[5,6]fulleren-3-yl)indolin-2-one (AIM 2). Yield 0.060 g (48 %); R_f 0.74 (Sorbfil, toluene : petroleum ether = 4 : 1); ^{1}H NMR (600.0

MHz, 298 K, $CS_2/CDCl_3$, δ, ppm): 8.30 (d, 1H, *J* = 7.8 Hz), 7.54-7.49 (m, 1H,), 7.24-7.20 (m, 1H), 7.15 (d, 1H, *J* 7.8 Hz), 4.02-3.98 (m, 2H), 2.03-1.94 (m, 2H), 1.22-1.19 (m, 3H); ^{13}C NMR (150.9 MHz, 298 K, $CS_2/CDCl_3$, δ, ppm): 168.27, 141.06, 129.21, 127.24, 123.71, 122.19, 108.45, 44.17, 41.77, 21.31, 11.69, fullerene moiety: 145.32, 145.22, 145.17, 144.89, 144.75, 144.71,144.48, 144.44, 144.23, 144.13, 143.98, 143.89, 143.86, 143.48, 143.34, 143.14, 142.86, 142.80, 142.70, 142.53, 142.11, 141.97, 141.92, 141.27, 140.67, 138.52, 75.07. IR (KBr, cm^{-1}): 2962, 2874, 1723, 1610, 1465, 1358, 1187, 742, 526; UV-VIS (CH_2Cl_2, nm, lg ε): 258 (5.25), 326 (4.60), 428 (3.47) (ribbon), 500 (3.31), 694 (2.38). MALDI-TOF-MS (matrix: DCTB) calcd for $C_{71}H_{11}NO$, 893.305; found 893.084.

1-*n*-Hexyl-3-(3-cyclopropane[1,9](C_{60}-*I*$_h$)[5,6]fulleren-3-yl)indolin-2-one (AIM 3). Yield 0.053 g (41 %); R_f 0.82 (Sorbfil, toluene : petroleum ether = 7 : 2); ^{1}H NMR (600.0 MHz, 298 K, $CS_2/CDCl_3$, δ, ppm): 8.32 (d, 1H, *J* = 7.7 Hz), 7.55-7.51 (m, 1H), 7.27-7.24 (m, 1H), 7.17 (d, 1H, *J* = 7.8 Hz), 4.05-4.01 (m 2H), 1.98-1.90 (m, 2H), 1.61-1.54 (m, 2H), 1.48-1.40 (m, 2H), 0.99-0.96 (m, 3H); ^{13}C NMR (150.9 MHz, 298 K, $CS_2/CDCl_3$, δ, ppm): 168.47, 141.99, 129.28, 125.00, 123.78, 122.33, 108.66, 40.76, 31.63, 27.67, 26.97, 22,84, 14.19, fullerene moiety: 145.35, 145.27, 144.97, 144.83, 144.55, 144.32, 144.20, 144.05, 143.95, 143.56, 143.39, 143.11, 142.87, 142.78, 142.62, 142.01, 141.35, 141.20, 141.13, 140.77, 138.67, 75.09. IR (KBr, cm^{-1}): 2916, 2844, 1723, 1609, 1465, 1357, 1181, 742, 525. UV-VIS (CH_2Cl_2' nm, lg ε): 257 (5.23), 329 (4.66),428 (3.37) (ribbon), 498 (3.23), 691 (2.28). MALDI-TOF-MS (matrix: DCTB) calcd for $C_{74}H_{17}NO$, 935.718; found 935.131.

1-*n*-Heptyl-3-(3-cyclopropane[1,9](C_{60}-*I*$_h$)[5,6]fulleren-3-yl)indolin-2-one (AIM 4). Yield 0.048 g (37 %); R_f 0.66 (Sorbfil, toluene : petroleum ether = 1 : 1); ^{1}H NMR (600.0 MHz, 298 K, $CS_2/CDCl_3$, δ, ppm): 8.30 (d, 1H, *J* = 7.8 Hz), 7.49-7.54 (m, 1H), 7.20-7.24 (m, 1H), 7.12 (d, 1H, *J*= 7.8 Hz), 4.00-4.03 (m, 2H), 1.97-1.90 (m, 2H), 1.61-1.49 (m,

4H), 1.40-1.28 (m, 4H), 0.98-0.96 (m, 3H); ^{13}C NMR (150.9 MHz, 298 K, $CS_2/CDCl_3$, δ, ppm): 168.25, 141.06, 129.19, 124.90, 123.70, 122.19, 108.46, 40.60, 31.88, 29.16, 27.73, 27.23, 22.91, 14.25, fullerene moiety: 145.31, 145.20, 144.94, 144.89, 144.71, 144.66, 144.47, 144.22, 144.12, 143.97, 143.86, 143.48, 143.33, 143.09, 142.86, 142.79, 142.70, 142.52, 142.07, 141.96, 141.91, 141.27, 140.66, 138.57, 75.05; IR (KBr, cm^{-1}): 2920, 2850, 1723, 1610, 1465, 1358, 1185, 745, 526. UV-VIS (CH_2Cl_2, nm, lg ε): 257 (5.13), 326 (4.61), 427 (3.32) (ribbon), 496 (3.18), 698 (2.28). MALDI-TOF-MS (matrix: DCTB) calcd for $C_{75}H_{19}NO$, 949.501; found 949.147.

1-*n*-Nonyl-3-(3-cyclopropane[1,9](C_{60}-*I*$_h$)[5,6]fulleren-3-yl)indolin-2-one (AIM 5). Yield 0.042 g (31 %); R_f 0.79 (Sorbfil, toluene : petroleum ether = 4 : 5); ^{1}H NMR (600.0 MHz, 298 K, $CS_2/CDCl_3$, δ, ppm): 8.28 (d, 1H, *J* = 7.8 Hz), 7.52-7.48 (m, 1H), 7.23-7.19 (m, 1H), 7.10 (d, 1H, *J* = 7.8 Hz), 4.02-3.98 (m, 2H), 1.96-1.89 (m, 2H), 1.60-1.48 (m, 4H), 1.36-1.33 (m, 8H), 0.95-0.92 (m, 3H); ^{13}C NMR (150.9 MHz, 298 K, $CS_2/CDCl_3$, δ, ppm): 168.34, 140.98, 129.15, 124.83, 123.64, 122.11, 108.37, 40.53, 31.95, 29.65, 29.48, 29.41, 27.73, 27.26, 22.96, 14.28, fullerene moiety: 145.29, 145.17, 144.87, 144.82, 144.68, 144.40, 144.36, 144.16, 144.05, 143.91, 143.81, 143.42, 143.27, 143.09, 142.80, 142.73, 142.63, 142.45, 142.10, 141.90, 141.86, 141.21, 140.48, 138.05, 75.02. IR (KBr, cm^{-1}): 2920, 2848, 1722, 1610, 1465, 1357, 1186, 742, 526. UV-VIS (CH_2Cl_2, nm, lg ε): 257 (5.25), 329 (4.77), 428 (3.39) (ribbon), 496 (3.26), 687 (2.29). MALDI-TOF-MS (matrix: DCTB) calcd. for $C_{77}H_{23}NO$, 977.178; found 977.415.

1-*n*-Decyl-3-(3-cyclopropane[1,9](C_{60}-*I*$_h$)[5,6]fulleren-3-yl)indolin-2-one (AIM 6). Yield 0.055 g (40 %); R_f 0.62 (Sorbfil, toluene : petroleum ether = 1 : 1); ^{1}H NMR (600.0 MHz, 298 K, $CS_2/CDCl_3$, δ, ppm): 8.32 (d, 1H, *J* = 7.8 Hz), 7.55-7.51 (m, 1H), 7.27-7.23 (m, 1H), 7.17 (d, 1H, *J* = 7.8 Hz), 4.04-4.00 (m, $2H_2$), 1.97-1.90 (m, 2H), 1.58-1.30 (m, 2H), 1.46-1.31 (m, 12H), 0.92-0.90 (m, 3H); ^{13}C NMR (150.9 MHz, 298 K, $CS_2/CDCl_3$, δ, ppm): 168.75, 142.09, 129.34, 125.10, 123.87, 122.42, 108.79, 41.99, 40.88, 31.96,

29.66, 29.44, 29.41, 27.74, 27.29, 22.84, 14.19, fullerene moiety: 145.45, 145.37, 145.10, 145.07, 145.04, 144.92, 144.89, 144.64, 144.61, 144.40, 144.27, 144.13, 143.07, 144.03, 143.64, 143.46, 143.23, 143.01, 142.95, 142.86, 142.70, 142.11, 141.30, 141.17, 140.83, 138.76, 75.22. IR (KBr, cm^{-1}): 2920, 2848, 1724, 1606, 1465, 1355, 1186, 742, 526. UV-VIS (CH_2Cl_2, nm), lg ε): 259 (5.19), 319 (4.69), 428 (3.34) (ribbon), 500 (3.20), 691(2.53). MALDI-TOF-MS (matrix: DCTB) calcd. for $C_{78}H_{25}NO$, 991.194; found 991.200.

1-*n*-Dodecyl-3-(3-cyclopropane[1,9](C_{60}-I_h)[5,6]fulleren-3-yl)indolin-2-one (AIM 7). Yield 0.059 g (41 %); R_f 0.72 (Sorbfil, toluene : petroleum ether = 1 : 1); ^{1}H NMR (600.0 MHz, 298 K, $CS_2/CDCl_3$, δ, ppm): 8.29 (d, 1H, J = 7.8 Hz), 7.53-7.49 (m, 1H), 7.24-7.20 (m, 1H), 7.13 (d, 1H, J = 7.8 Hz), 4.02-3.99 (m, $2H_2$), 1.99-1.89 (m, 2H), 1.60-1.53 (m, 2H), 1.51-1.44 (m, 2H) 1.35-1.30 (m, 14H), 0.94-0.91 (m, 3H,); ^{13}C NMR (150.9 MHz, 298 K, $CS_2/CDCl_3$, δ, ppm): 168.35, 141.07, 129.20, 124.92, 123.70, 122.20, 108.49, 41.80, 40.63, 32.01, 29.78, 29.76, 29.75, 29.71, 29.48, 27.73, 27.27, 22.96, 14.27, fullerene moiety: 145.32, 145.22, 144.94, 144.90, 144.76, 144.71, 144.47, 144.43, 144.23, 144.12, 143.97, 143.88, 143.48, 143.33, 143.10, 142.86, 142.80, 142.70, 142.53, 142.07, 141.97, 141.92, 141.27, 141.04, 140.66, 138.58, 75.07. IR (KBr, cm^{-1}): 2919, 2848, 1723, 1611, 1465, 1357, 742, 526. UV-VIS (CH_2Cl_2, nm, lg ε): 257 (4.96), 329 (4.42), 431 (3.44) (ribbon), 500 (3.29), 694 (2.44). MALDI-TOF-MS (matrix: DCTB) calcd for $C_{80}H_{29}NO$, 1019.279; found 1019.225.

1-*n*-Tetradecyl-3-(3-cyclopropane[1,9](C_{60}-I_h)[5,6]fulleren-3-yl)indolin-2-one (AIM 8). Yield 0.045 g (31 %), R_f 0.38 (Sorbfil, toluene : petroleum ether = 3 : 2); ^{1}H NMR (600.0 MHz, 298 K, $CS_2/CDCl_3$, δ, ppm): 8.28 (d, 1H, J= 7.8 Hz), 7.50-7.46 (м, 1H), 7.21-7.17 (m, 1H), 7.10 (d, 1H, J = 7.8 Hz), 4.00-3.97 (m, 2H), 1.95-1.87 (m, 2H), 1.60-1.47 (m, 4H), 1.36-1.30 (m, 18H), 0.94-0.91 (m, 3H); ^{13}C NMR (150.9 MHz, 298 K, $CS_2/CDCl_3$, δ, ppm): 167.66, 140.93, 129.09, 124.76, 123.58, 122.00, 108.25, 41.86,

40.64, 32.11, 29.79, 29.77, 29.76 (br), 29.71 (br), 29.47, 29.48, 27.73, 27.27, 22.96, 14.27, fullerene moiety: 145.26, 145.12, 144.81, 144.76, 144.67, 144.62, 144.60, 144.35, 144.29, 144.10, 143.99, 143.86, 143.77, 143.74, 143.36, 143.22, 143.07, 142.73, 142.68, 142.57, 142.39, 142.15, 141.85, 141.81, 141.16, 140.89, 140.50, 138.43, 74.99. IR (KBr, cm^{-1}): 2919, 2847, 1721, 1610, 1463, 1355, 1185, 741, 526. UV-VIS (CH_2Cl_2, nm; lg ε: 259 (5.12), 330 (4.55), 428 (3.33) (ribbon), 500 (3.19), 694 (2.27). MALDI-TOF-MS (matrix: DCTB) calcd for $C_{82}H_{33}NO$, 1047.256; found 1047.833.

1-*n*-Hexadecyl-3-(3-cyclopropane[1,9](C_{60}-*I*$_h$)[5,6]fulleren-3-yl)indolin-2-one (AIM 9). Yield 0.046 g (33 %). R_f 0.68 (Sorbfil, toluene : petroleum ether = 1 : 1). ^{1}H NMR (600.0 MHz, 298 K, $CS_2/CDCl_3$, δ, ppm): 8.30 (d, 1H, J = 7.8 Hz), 7.53-7.51 (m, 1H), 7.24-7.22 (m, 1H), 7.15 (d, 1H, J = 7.8 Hz), 4.03-4.00 (m, 2H), 1.95-1.90 (m, 2H), 1.58-1.53 (m, 2H), 1.49-1.44 (m, 2H), 1.38-1.29 (m, 22H), 0.93-0.91 (m, 3H); ^{13}C NMR (150.9 MHz, 298 K, $CS_2/CDCl_3$, δ, ppm): 167.46, 141.07, 129.23, 124.94, 123.73, 122.26, 108.56, 41.79, 40.67, 32.03, 29.82 (br), 29.49 (br), 27.71, 27.28, 22.96, 14.29, fullerene moiety: 145.31, 145.23, 144.97, 144.92, 144.78, 144.73, 144.50, 144.25, 144.14, 143.99, 143.89, 143.50, 143.34, 143.07, 142.88, 142.81, 142.72, 142.56, 141.99, 141.93, 141.30, 141.11, 140.70, 138.61, 75.05. IR (KBr, cm^{-1}): 2921, 2849, 1723, 1610, 1464, 1356, 1186, 741, 526. UV-VIS (CH_2Cl_2, nm, lg ε: 257 (5.14), 326 (4.64), 428 (3.34) (ribbon), 500 (3.21), 691 (2.27). MALDI-TOF-MS (matrix: DCTB) found 1076.402 (calcd for $C_{84}H_{37}NO$ 1076.402).
